# Frontal Base Mixed Pial‐Dural Arteriovenous Malformations: A Distinct Entity Requiring Differentiated Treatment From Anterior Cranial Fossa Dural Arteriovenous Fistulas

**DOI:** 10.1002/brb3.71099

**Published:** 2025-12-07

**Authors:** Zhijie Jiang, Si Hu, Guoqiang Zhang, Jingwei Zheng, Fei Liu, Xudan Shi, Chenhan Ling, Jing Xu, Jun Yu, Liang Xu

**Affiliations:** ^1^ Department of Neurosurgery Second Affiliated Hospital School of Medicine Zhejiang University Hangzhou Zhejiang China; ^2^ Clinical Research Center for Neurological Diseases of Zhejiang Province Hangzhou Zhejiang China; ^3^ State Key Laboratory of Transvascular Implantation Devices Hangzhou Zhejiang China; ^4^ Department of Neurosurgery Affiliated Huzhou FuYin Hospital of Huzhou University Huzhou Zhejiang China; ^5^ Department of Neurocritical Care Second Affiliated Hospital School of Medicine Zhejiang University Hangzhou Zhejiang China; ^6^ Department of Anesthesiology Second Affiliated Hospital School of Medicine Zhejiang University Hangzhou Zhejiang China

## Abstract

**Background:**

Frontal base mixed pial‐dural arteriovenous malformations (MPD‐AVMs) are rare intracranial vascular malformations with both pial and dural components. Although they share some angioarchitectural similarities with anterior cranial fossa dural arteriovenous fistulas (ACF‐DAVFs), the two represent different pathological processes, as ACF‐DAVFs are supplied exclusively by dural arteries. This study provides an overview of frontal base MPD‐AVMs, highlighting their differences from ACF‐DAVFs, and discusses the therapeutic implications of their distinct angioarchitectural features.

**Methods:**

This is a single‐center case series study conducted between January 2018 and December 2024. The data of 11 patients who underwent endovascular treatment for frontal base MPD‐AVMs and 29 patients diagnosed with ACF‐DAVFs were retrospectively reviewed.

**Results:**

In patients diagnosed with MPD‐AVMs, all lesions were supplied by the anterior ethmoidal artery (AEA) and orbitofrontal artery (OFA). Flow‐related aneurysms in the OFA were identified in seven patients (7/11, 63.6%), with three presenting with hemorrhagic events. Treatment approaches included transarterial embolization (TAE) in eight patients, with one requiring additional transvenous embolization (TVE). Primary TVE was employed in three patients, including two hemorrhagic patients who underwent staged treatment with initial aneurysm embolization. Complete obliteration was achieved in 72.7% of cases (8/11), although one patient experienced postoperative hemorrhage. In patients presenting with ACF‐DAVFs, venous aneurysm (18/29, 62.1%) might represent a risk factor for bleeding (13/29, 44.8%) (*p* = 0.003). In the MPD‐AVM group, venous aneurysms were detected in five patients (5/11, 45.5%), but none of whom experienced hemorrhage.

**Conclusion:**

Frontal base MPD‐AVMs represent distinct vascular anomalies from ACF‐DAVFs, often featuring anterior cerebral artery branch involvement. Tailored multi‐arterial endovascular strategies are crucial for optimizing outcomes and minimizing complications. Further studies with larger cohorts are essential to validate these observations and refine treatment guidelines.

## Graphical abstract

Frontal lobe MPD‐AVMs are characterized by pial‐dural vascular supply and anterior cerebral artery branch involvement, distinguishing them from ACF‐DAVFs. Tailored multi‐arterial endovascular strategies are critical for effective management, emphasizing the need for lesion‐specific approaches to optimize therapeutic outcomes and mitigate risks.

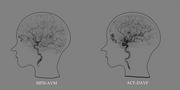

## 1 Introduction

Dural arteriovenous fistula (DAVF) is a complex neurovascular disorder characterized by direct shunting between dural arteries and venous drainage systems. Due to its intricate angioarchitecture, DAVF can present diagnostic and management challenges, even for experienced clinicians. Most DAVFs are typically supplied by the dural arteries, although rare cases, particularly those involving the tentorium, have been reported with pial arterial contributions (Bhogal et al. 2015; Carnevale et al. 2022; Hetts et al. 2017; Maki et al. 2021). Among the various types of DAVF, anterior cranial fossa DAVF (ACF‐DAVF) is a rare subtype, comprising only 10% of all DAVFs (Limbucci et al. 2018). These are typically fed exclusively by dural arteries. To date, only two cases of ACF‐DAVFs with pial arterial supply have been documented (Tsutsumi et al. 2009; Yamano et al. 2020). These pial supplies generally originate from the anterior cerebral artery (ACA), with the orbitofrontal artery (OFA) branches being the most common source (Maki et al. 2021; Jimbo et al. 2010). Although several case series have described ACF‐DAVFs with ACA involvement (Su et al. 2024; Tahon et al. 2008), some researchers argue that the angioarchitecture of these cases deviates from classical ACF‐DAVFs and may represent a distinct entity—frontal base mixed pial‐dural arteriovenous malformations (MPD‐AVMs) (Jimbo et al. 2010). Frontal base MPD‐AVM is defined as a rare but distinct subgroup of frontal lobe arteriovenous malformations (AVMs), receiving concurrent supply from pial and dural arteries. Two anatomical patterns are recognized based on the location of the shunting point: one with the nidus situated within the frontal lobe parenchyma, and the other with the primary shunting point located primarily within the dura mater (Jimbo et al. 2010).

While DAVFs and AVMs are fundamentally different vascular pathologies, they may be radiologic mimics. Accurate differentiation between front base MPD‐AVMs and ACF‐DAVFs in the anterior cranial fossa is essential. This distinction is essential, as the treatment strategies for these two conditions differ significantly. Misdiagnosis or inappropriate management could lead to serious, potentially devastating consequences.

In this report, we present our single‐center experience with frontal base MPD‐AVMs, focusing on their angioarchitecture and contrasting management strategies with ACF‐DAVFs. We aim to propose a practical diagnostic and therapeutic approach for this rare and challenging condition.

## 2 Methods

### 2.1 Patient Enrollment and Data Collection (Figure S1)

This retrospective study was conducted at our institution, encompassing 11 consecutive patients who underwent endovascular treatment for frontal base vascular malformations between January 2018 and December 2024. Baseline demographic and clinical data, including age, sex, presenting symptoms, Glasgow Coma Scale (GCS) score upon admission, modified Rankin Scale (mRS) score, hemorrhagic presentation, and angiographic results, are summarized in Table 1. A comparison group consisting of 29 patients with ACF‐DAVF was also included in the analysis (Table S1). All clinical and radiological data were independently reviewed by two experienced neurointerventionalists to ensure the accuracy and reliability.

**TABLE 1:** Baseline and clinical characteristics of patients.

No.	Initial symptoms	Type	Hemorrhage	Flow‐related aneurysm	Venous aneurysm	Feeders	Venous drainage	Approach	Route	Coil	Onyx	IAR	Complications	GCS		mRS	
														Pre.	Post.	Pre.	Post.
1	Headache	I	Yes	Yes	No	Rt‐AEA and OFA	CV	TAE	Rt‐AEA	0	0.4mL	InCo.	None	15	15	2	0
2^a^	Headache	I	Yes	Yes	No	Rt‐AEA and OFA	CV	TVE	SSS‐CV	2	1.8mL	Co.	None	15	15	4	0
3	Asymptomatic	I	No	No	No	Bi‐AEA and OFA	CV	TAE + TVE	Rt‐OFA and SSS‐CV	0	3.2mL	Co.	None	15	15	0	0
4	Asymptomatic	I	No	Yes	Yes	Rt‐AEA and OFA	CV	TVE	SSS‐CV	2	1.2mL	Co.	Post‐embolization hemorrhage	15	15	0	1
5	Headache	I	No	Yes	No	Bi‐AEA and OFA	CV	TAE	Lt‐OFA and AEA	0	2mL	Co.	None	15	15	0	0
6	Asymptomatic	I	No	Yes	No	Lt‐AEA and OFA	CV	TAE	Lt‐OFA	0	2mL	Co.	None	15	15	0	0
7	Asymptomatic	I	No	Yes	Yes	Rt‐AEA and OFA	CV	TAE	Rt‐OFA and AEA	0	0.5mL	Co.	None	15	15	0	0
8	Dizziness	II	No	No	Yes	Lt‐AEA and OFA	CV	TAE	Lt‐OFA and AEA	0	2.2mL	Co.	None	15	15	0	0
9^a^	Headache	I	Yes	Yes	No	Lt‐AEA and OFA	CV	TVE	SSS‐CV	1	0.8mL	Co.	None	15	15	2	0
10	Asymptomatic	II	No	No	Yes	Lt‐AEA and OFA, Bi‐IMA	CV	TAE	Lt‐OFA and AEA	0	1.0mL	InCo.	None	15	15	0	0
11	Asymptomatic	II	No	No	Yes	Bi‐AEA and IMA, Rt‐OFA	CV	TAE	Bi‐AEA and Lt‐OFA	0	1.3mL	InCo.	None	15	15	0	0

*Note*: A total of 11 patients were included, with a male‐to‐female ratio of 8:3 and a mean age of 61.2 ± 12.5 years (range: 47–80 years).

Abbreviations: AEA, anterior ethmoidal artery; Bi, bilateral; Co., complete; CV, cortical vein; GCS, Glasgow Coma Scale; IAR, immediate angiographic results; InCo., incomplete; Lt, left; mRS, modified Rankin Scale; OFA, orbitofrontal artery; Post., postoperative; Pre., preoperative; Rt, right; SSS, superior sagittal sinus; TAE, transarterial embolization; TVE, transvenous embolization.

^a^The two patients were admitted with SAH due to rupture of a flow‐related aneurysm. For each patient, staged endovascular treatment was performed, with aneurysm embolization in the first stage and frontal lobe MPD‐AVM embolization in the second stage.

### 2.2 Analysis of Angioarchitecture

All patients underwent selective six‐vessel cerebral digital subtraction angiography (DSA) to accurately delineate the angioarchitecture of the vascular malformations. High‐resolution computed tomography (CT) and magnetic resonance imaging (MRI) were implemented as complementary diagnostic tools. Detailed characteristics of the malformations, including feeding arteries, draining veins, and the presence of flow‐related or venous aneurysms, were meticulously documented. Patients diagnosed with MPD‐AVMs were classified into two types: Type I MPD‐AVM (parenchymal nidus) and Type II MPD‐AVM (dural nidus) (Maki et al. 2021; Yamano et al. 2020; Jimbo et al. 2010; Yamamoto et al. 2022). This proposed classification is introduced here for terminological clarity. The angiographic findings were independently evaluated by at least two senior physicians to ensure diagnostic reliability and accuracy. Particular attention was given to flow‐related aneurysms of the OFA and venous aneurysms, as these might be associated with a higher risk of hemorrhagic events.

### 2.3 Clinical Outcomes and Follow‐Up

Post‐embolization assessments, including immediate DSA and intraoperative CT, were performed to evaluate treatment efficacy and identify potential complications. The immediate treatment outcomes were categorized using standardized criteria: complete embolization, defined as total obliteration of the AVM with no residual nidus, and incomplete embolization, defined as any persistent arteriovenous shunting.

Neurological status was assessed using the mRS at discharge and during follow‐up visits. Surveillance DSA was recommended 6 months post‐treatment for all eligible patients. For those who declined angiographic evaluation, follow‐up was conducted using high‐resolution CT, CT angiography, and MRI with time‐of‐flight sequences.

### 2.4 Treatment Strategy

In the MPD‐AVM group, the transarterial approach was preferentially employed. The transarterial embolization (TAE) was performed using a standard technique, with a 5‐ or 6‐Fr guiding catheter positioned within the internal carotid artery (ICA). Under roadmap guidance, the microcatheter was precisely advanced into the feeding artery. Upon confirmation of its position at the nidus or shunting point by microangiography, Onyx (Medtronic) was injected slowly and carefully, with particular attention to minimizing reflux. To achieve complete embolization, multi‐arterial approach might be considered. In cases with associated flow‐related aneurysms, the initial treatment strategy might involve targeted aneurysm embolization alone. We do not recommend the routine use of transvenous embolization (TVE) in cases of MPD‐AVM.

In the ACF‐DAVF group, endovascular treatment utilized either a TAE or TVE strategy depending on the angioarchitecture. For TAE, A 5‐ or 6‐Fr guiding catheter was introduced into the internal or external carotid artery. Under roadmap assistance, the microcatheter was navigated to the feeding artery and further advanced to the fistula point. Onyx (Medtronic) was then injected slowly under fluoroscopic monitoring to limit reflux until complete fistula occlusion was confirmed angiographically. For TVE, access was established via a 6‐Fr sheath in the right femoral vein and a 5‐Fr sheath in the right femoral artery. Diagnostic angiography was first performed through the arterial feeder for roadmapping. A 6‐Fr intermediate catheter was advanced to the superior sagittal sinus after navigating the internal jugular vein bulb. Using a “wire‐loop” technique with a U‐shaped microguidewire, the microcatheter was directed to the fistula. When feasible, two microcatheters were advanced into the cortical vein. Coils were initially deployed using a retrograde pressure cooker technique to form a scaffold, followed by controlled injection of Onyx—initially slow, then gradually accelerated—while strictly limiting the total volume to prevent non‐target embolization.

### 2.5 Statistical Analysis

Statistical analyses were conducted using IBM SPSS Statistics for Windows, version 25.0 (IBM Corporation, Armonk, NY, USA). Continuous variables were presented as mean ± standard deviation, while categorical variables were expressed as frequencies and percentages. Categorical variables were analyzed using chi‐square test or Fisher's exact test, as appropriate. All statistical tests were two‐tailed, and a *p* value < 0.05 was considered statistically significant.

## 3 Results

### 3.1 Patient Demographics

A total of eleven patients were included in this study, with a male‐to‐female ratio of 8:3. The mean age was 61.2 ± 12.5 years (range: 47–80 years). Six patients were asymptomatic and were identified incidentally during routine medical examinations. The remaining patients presented with clinical symptoms, including dizziness in one patient and headache in the others. Among the patients with headaches, three experienced hemorrhagic events, which were confirmed by emergent CT scans upon admission: one with a frontal lobe hematoma and the other with subarachnoid hemorrhage (SAH). No hemorrhagic events were observed in the asymptomatic patients.

Regarding preoperative neurological status, eight non‐hemorrhagic patients had a GCS score of 15 and an mRS score of 0. Of the three hemorrhagic patients, two presented with mild symptoms (GCS 15, mRS 2): one with frontal lobe hematoma and another with SAH. The third patient, who also had SAH, maintained consciousness but exhibited significant functional impairment (GCS 15, mRS 4).

### 3.2 Angioarchitecture

All 11 patients in the MPD‐AVM group exhibited both dual and pial arterial blood supply. Eight patients were classified as Type I, and the remaining three as Type II. The primary feeders included the anterior ethmoidal artery (AEA) and branches of the ACA, particularly the OFA. Feeders from the internal maxillary artery (IMA) were identified in two patients. Preoperative DSA revealed unilateral blood supply in seven patients and bilateral supply in the remaining four. Flow‐related aneurysms in the OFA were identified in seven patients and were likely the cause of the three hemorrhagic events, with no other lesion components contributing to the hemorrhage. Venous aneurysms were observed in five asymptomatic patients. While three patients were confirmed not to have a nidus, the remaining eight patients all demonstrated a distinguishable nidus on their angiograms.

In the ACF‐DAVF group, the AEA was the primary feeder in the majority of the cases (28/29, 96.6%), with the exception of one patient whose lesion was supplied by bilateral facial arteries. In addition, the superficial temporal artery, middle meningeal artery, and IMA were also observed to contribute to the blood supply in certain patients. No pial supply was observed in any of the patients. Venous aneurysms were detected in 18 patients (18/29, 62.1%) and may represent a potential risk factor for hemorrhage, which was observed in 13 patients (13/29, 44.8%) (*p* = 0.003). Detailed angioarchitectural characteristics are summarized in Table 1.

### 3.3 Treatment Strategies and Outcomes

In the MPD‐AVM group, treatment strategies initially focused on embolization of the flow‐related aneurysms in the three hemorrhagic patients. In one case, an attempt was made to obliterate the AVM using Onyx via a transarterial approach through the AEA branches; however, complete embolization was not achieved. The other two hemorrhagic patients underwent a staged treatment approach: flow‐related aneurysm coiling was performed initially to reduce the risk of recurrent bleeding, followed by complete obliteration of the nidus via a transvenous approach (TVE).

Among the eight non‐hemorrhagic patients, seven underwent TAE as the primary treatment strategy. Complete embolization was achieved in one patient via uni‐arterial access through the OFA and in three patients via multi‐arterial access involving the AEA and OFA. In one additional case, TAE was complemented by a subsequent TVE to achieve complete nidus obliteration. The remaining non‐hemorrhagic patient underwent TVE as the initial treatment. While the postoperative angiogram demonstrated complete obliteration of the nidus, the patient experienced a postoperative hemorrhage, necessitating urgent decompressive craniectomy and hematoma evacuation. The overall immediate complete obliteration rate following endovascular treatment of MPD‐AVMs was 72.7% (8/11), with a procedure‐related complication rate of 9.1% (1/11).

In the ACF‐DAVF group, single‐stage embolization was performed on all patients. The immediate occlusion rate achieved with TVE was significantly higher than that of TAE (15/15, 100% vs. 10/14, 71.4%; *p* = 0.042). Post‐embolization complications occurred in two patients (6.9%, 2/29), specifically a thromboembolism in one patient and a microcatheter fracture in the other. A summary of the surgical techniques and embolization materials used is provided in Table 1.

### 3.4 Patient Outcomes and Follow‐Up

All patients diagnosed as MPD‐AVM were followed up with DSA or MRA imaging. No radiological evidence of residual or recurrent lesions was found during follow‐up, except for three patients who had incomplete embolization. Regarding neurological status, only the patient who experienced postoperative hemorrhage after the initial TVE had an mRS score of 1 upon discharge. All other patients recovered well, with no significant neurological deficits at the latest follow‐up.

### 3.5 Presentation of Typical Cases

#### 3.5.1 Case 1: Multi‐Arterial TAE Strategy in Frontal MPD‐AVM (Figure 1)

A middle‐aged patient presented with recurrent headaches lasting over 1 month. Preadmission brain MRI revealed a vascular malformation in the anterior cranial fossa. DSA demonstrated a frontal base MPD‐AVM with bilateral ACA and AEA blood supply. A flow‐related aneurysm was observed at the origin of the OFA, with aneurysmal dilatation at the distal segment. Intraoperative angiogram confirmed the presence of a nidus after catheterization, followed by complete embolization using a multi‐arterial approach, without affecting any critical arterial branches. The patient was discharged 3 days post‐operation with significant relief of her headache and no neurological deficits. Follow‐up angiography confirmed stable embolization of the lesion.

#### 3.5.2 Case 2: Efficacy of Hybrid Embolization Strategy in Frontal MPD‐AVM (Figure 2)

An elderly patient presented with sudden‐onset severe headache, accompanied by nausea and vomiting. Emergency non‐contrast CT revealed extensive SAH and a frontal lobe hematoma. DSA revealed an anterior cranial fossa MPD‐AVM supplied by the right OFA, with multiple flow‐related aneurysms, which were deemed responsible for the bleeding event. A staged management approach was implemented, starting with embolization of the ruptured aneurysms and the parent artery. This was followed by secondary embolization via a transvenous approach using the “reverse pressure cooker technique” 40 days later (Chapot et al. 2014). Follow‐up imaging demonstrated no recurrence of the nidus.

#### 3.5.3 Case 3: Pitfalls of Isolated TVE in Frontal MPD‐AVM: Lessons From a Complicated Case (Figure 3)

An adult patient was incidentally diagnosed with a frontal base vascular malformation during a routine health examination. DSA revealed a lesion with dual blood supply, from both the right AEA and pial supply from the right OFA, along with a flow‐related aneurysm at the ophthalmic segment of the right ICA. The patient initially underwent TVE. Although immediate post‐procedural angiography showed complete obliteration of the nidus, the patient developed a massive intraparenchymal hemorrhage in the right frontal lobe, necessitating urgent decompressive craniectomy and hematoma evacuation. The patient was discharged with an mRS score of 1 and showed no neurological deficits or radiological residuals of the lesion at long‐term follow‐up.

## 4 Discussion

While DAVFs can broadly be categorized as a subtype of AVM, it is essential to carefully analyze the angioarchitecture of intracranial vascular malformations, as treatment strategies differ significantly. DAVFs are usually considered to be supplied by merely dural feeders. The debate continues regarding whether lesions with pial feeders, especially those located in the anterior cranial fossa, should be classified as DAVFs or MPD‐AVMs. With advancements in neuroimaging and neurointerventional techniques, a deeper understanding of the angioarchitecture and therapeutic strategies for ACF‐DAVFs has been achieved. These rare intracranial vascular malformations account for approximately 10% of all DAVFs and are typically supplied exclusively by dural vessels (Limbucci et al. 2018; Jimbo et al. 2010; Kwon et al. 2005). ACF‐DAVFs with pial arterial supply are exceptionally rare, with only two cases documented in the literature, involving blood supply from branches of the OFA and the posterior pial orbital artery (PPOA), respectively (Tsutsumi et al. 2009; Yamano et al. 2020). This unique vascular pattern has sparked significant academic debate, with Jimbo et al. (2010) suggesting that lesions with pial supply should be reclassified as MPD‐AVMs of the frontal base rather than DAVFs.

Frontal base MPD‐AVMs represent a rare subset of vascular malformations, with an even lower incidence than ACF‐DAVFs. To date, only 14 sporadic cases have been reported (Table S2), precluding accurate epidemiological assessment (Yamano et al. 2020; Jimbo et al. 2010). These lesions are characterized by a dual vascular supply from both dural and pial vessels. The dural component typically derives from the AEA, while the pial supply predominantly originates from the OFA, anterior falcine artery, PPOA, frontobasal artery, and other ACA branches. Unlike typical AVMs in other locations, frontal base MPD‐AVMs may not exhibit a clearly delineated nidus on angiograms, complicating their differentiation from ACF‐DAVFs. This phenomenon can be attributed to the presence of arteriovenous fistulous components within the lesion, which may lead to a steal phenomenon, potentially obscuring the nidus. After flow reduction through embolization of the shunting structure, microcatheter angiography may reveal previously concealed nidus components. This observation underscores the importance of staged embolization strategies, particularly via a multi‐arterial approach, in complex vascular malformations. Initial flow reduction may unmask additional pathological components requiring treatment.

MPD‐AVMs can be classified into two distinct subtypes based on their angioarchitectural features: Type I lesions, which have a pial‐supplied nidus within the frontal cortical parenchyma, and Type II lesions, which exhibit physiological anastomoses between pial and dural vessels at the dural interface (Maki et al. 2021; Yamano et al. 2020; Jimbo et al. 2010; Yamamoto et al. 2022). These subtypes demonstrate significant heterogeneity in their pathophysiological mechanisms, clinical manifestations, and therapeutic strategies. Type I lesions share morphological and hemodynamic features with traditional AVMs, whereas Type II lesions exhibit characteristics more akin to DAVFs, which typically occur at the tentorium via physiological pial‐dural anastomoses, specifically the Davidoff and Schechter arteries (Carnevale et al. 2022; Maki et al. 2021; Jimbo et al. 2010). The present study included seven Type I lesions and one Type II lesion. The clinical presentation patterns may serve as distinguishing features between these entities. While typical ACF‐DAVFs frequently present with frontal lobe hematoma upon rupture, frontal lobe MPD‐AVMs are characteristically associated with flow‐related aneurysms of the OFA, potentially manifesting as acute SAH following aneurysmal rupture (Table 2). Furthermore, hemorrhage from the nidus or dilated draining veins may present as intracerebral hematomas, creating a clinical presentation that parallels ruptured DAVFs. These distinct hemorrhagic patterns, combined with comprehensive neuroimaging findings, may facilitate accurate differentiation between these vascular entities.

**TABLE 2:** Comparison between frontal base MPD‐AVM and ACF‐DAVF.

Feature	MPD‐AVM	ACF‐DAVF
Arterial supply	Dual supply from both pial (e.g., OFA) and dural (e.g., AEA) arteries	Supply exclusively from dural arteries (primarily AEA)
Key angioarchitectural features	Characterized by a parenchymal nidus (Type I) or a dural‐based nidus (Type II); sometimes accompanied by flow‐related aneurysms on pial feeders (e.g., OFA) in a subset of patients	Incidence of venous aneurysms; no nidus, single fistulous point
Primary hemorrhagic risk factor	Nidus rupture (analogous to conventional AVMs); additionally, SAH may result specifically from flow‐related aneurysm rupture	Venous aneurysm is a high‐risk factor for rupture; aggressive cortical venous drainage also confers significant hemorrhage risk (Xu et al. 2025)
First‐line endovascular approach	Transarterial embolization (TAE), sometimes requiring a multi‐arterial strategy	Transvenous embolization (TVE) is highly effective (Xu et al. 2025)
Pitfall to avoid	Isolated TVE carries a high risk of post‐procedural rebleeding due to incomplete nidus occlusion	For ACF‐DAVF, TAE is associated with a lower success rate compared to TVE and carries a risk of non‐target embolization (e.g., central retinal artery occlusion) (Xu et al. 2025)

Therapeutic strategies for MPD‐AVMs have evolved considerably, encompassing both microsurgical approaches and endovascular techniques. Microsurgical resection, historically considered the standard treatment, generally achieves favorable outcomes due to the superficial location of these lesions. The surgical approach varies depending on the subtype: Type I MPD‐AVMs require complete nidus excision, while Type II lesions can often be managed effectively by selective disconnection of the arteriovenous shunting points alone (Maki et al. 2021; Yamano et al. 2020; Jimbo et al. 2010; Yamamoto et al. 2022). In hemorrhagic presentations, microsurgical intervention offers the additional advantage of concurrent hematoma evacuation. While conventional microsurgical approaches carry inherent risks, such as intraoperative hemorrhage, postoperative infections, iatrogenic frontal lobe injury, and cerebrospinal fluid leakage, surgery remains the first‐line treatment option in characteristic patients.

Recent advances in endovascular techniques and materials have established alternative therapeutic options, underpinned by a comprehensive understanding of the angioarchitecture and pathophysiology of these lesions. For Type I MPD‐AVMs, endovascular treatment follows similar principles to those used for traditional AVMs, requiring complete occlusion of major and collateral feeding arteries and total nidus obliteration (Maki et al. 2021; Jimbo et al. 2010; Puthuran et al. 2024). It is recommended to use a multi‐arterial approach as the first‐line strategy for embolization of these lesions. This approach, utilizing multiple arterial access points, provides several advantages: it allows for more thorough treatment of the pathological vasculature, potentially reduces the risk of incomplete occlusion and may help avoid complications associated with isolated TVE. In addition, staged multi‐arterial embolization enables better control of hemodynamic changes during the procedure and may facilitate safer and more complete obliteration of the lesion. In cases with flow‐related aneurysms, which often represent the primary source of hemorrhage, initial intervention can focus on embolizing the aneurysm to achieve immediate hemorrhage control, without requiring single‐session complete obliteration of the lesion.

Although TVE has demonstrated promising therapeutic efficacy in the treatment of ACF‐DAVF, it is crucial to note that isolated TVE carries substantial risks and should be considered a potential pitfall in the management of MPD‐AVMs (Table 2). Venous outflow obstruction without complete nidus embolization can lead to hazardous postoperative rebleeding, as demonstrated in Case 3 of the present cohort. In contrast, TVE can be considered a salvage treatment if TAE cannot achieve complete lesion obliteration. However, when TVE is used, it is crucial to achieve complete occlusion of both the draining veins and nidus in a single session. Controlled systemic hypotension, balloon assistance to reduce perfusion pressure, and preliminary arterial embolization for flow modification may help lower the risk of complications during TVE.

### 4.1 Limitations

The retrospective analysis conducted at a single institution may introduce potential biases inherent in data collection and interpretation. Furthermore, the statistical power of our findings is limited by the modest cohort size and non‐randomized patient allocation process, which depended on individualized vascular morphological assessments and clinician discretion rather than standardized randomization protocols. These methodological considerations emphasize the imperative for multicenter prospective investigations with balanced enrollment criteria to corroborate our findings.

## 5 Conclusion

Frontal base MPD‐AVMs are distinct vascular entities from ACF‐DAVFs, often characterized by the involvement of ACA branch supply. These lesions require significantly different therapeutic approaches compared to ACF‐DAVFs. Misunderstanding of the angioarchitecture can lead to inappropriate management and potentially life‐threatening complications. Further studies with larger patient cohorts are needed to validate these preliminary findings and establish more definitive treatment guidelines.

## Author Contributions

Zhijie Jiang, Si Hu, Guoqiang Zhang, and Liang Xu had full access to all the study data and take responsibility for the integrity of the data. Concept and design: Zhijie Jiang, Si Hu, Liang Xu, and Jun Yu. Acquisition, analysis, or interpretation of data: Si Hu, Zhijie Jiang, Fei Liu, Xudan Shi, Chenhan Ling, and Liang Xu. Drafting of the manuscript: Si Hu, Zhijie Jiang, and Liang Xu. Critical revision of the manuscript for important intellectual content: Si Hu, Zhijie Jiang, Jingwei Zheng, and Liang Xu. Statistical analysis: Zhijie Jiang, Jingwei Zheng, and Liang Xu. Funding acquisition: Jingwei Zheng, Jun Yu, and Liang Xu. Administrative, technical, or material support: Jing Xu, Jun Yu, and Liang Xu. Supervision: Liang Xu.

## Funding

This study was supported by the National Natural Science Foundation of China (82301454) to Jingwei Zheng, the National Natural Science Foundation of China (81971097) to Jun Yu, the Zhejiang Provincial Natural Science Foundation of China (LY22H090023) to Jun Yu, and the Medical Health Science and Technology Project of Zhejiang Provincial Health Commission (2022RC168) to Liang Xu.

## Ethics Statement

The study was conducted in accordance with the Declaration of Helsinki and approved by the institutional ethics committee (Approval ID: 20241476, The Second Affiliated Hospital of Zhejiang University, School of Medicine). The requirement for informed consent was waived due to the retrospective nature of the study.

## Conflicts of Interest

The authors declare no conflicts of interest.

6

**FIGURE 1:**
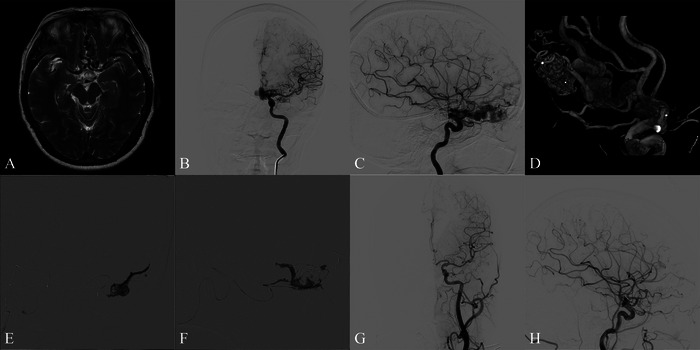
The patient was admitted with headaches. A T2‐weighted MRI revealed an abnormal flow void at the anterior cranial fossa (A). Cerebral angiography was performed, with anteroposterior (B) and lateral (C) views of the left internal carotid artery showing a vascular malformation in the anterior cranial fossa, supplied by branches of the anterior cerebral and ethmoidal arteries. 3D reconstruction clearly visualized the nidus (D), confirming the diagnosis of an arteriovenous malformation (AVM). Onyx‐18 embolization was carried out through multiple arterial feeders (orbitofrontal artery and ethmoidal artery) under fluoroscopic guidance (E, F). Post‐procedure angiography in anteroposterior (G) and lateral (H) views confirmed complete obliteration of the AVM, with preservation of normal branches.

**FIGURE 2:**
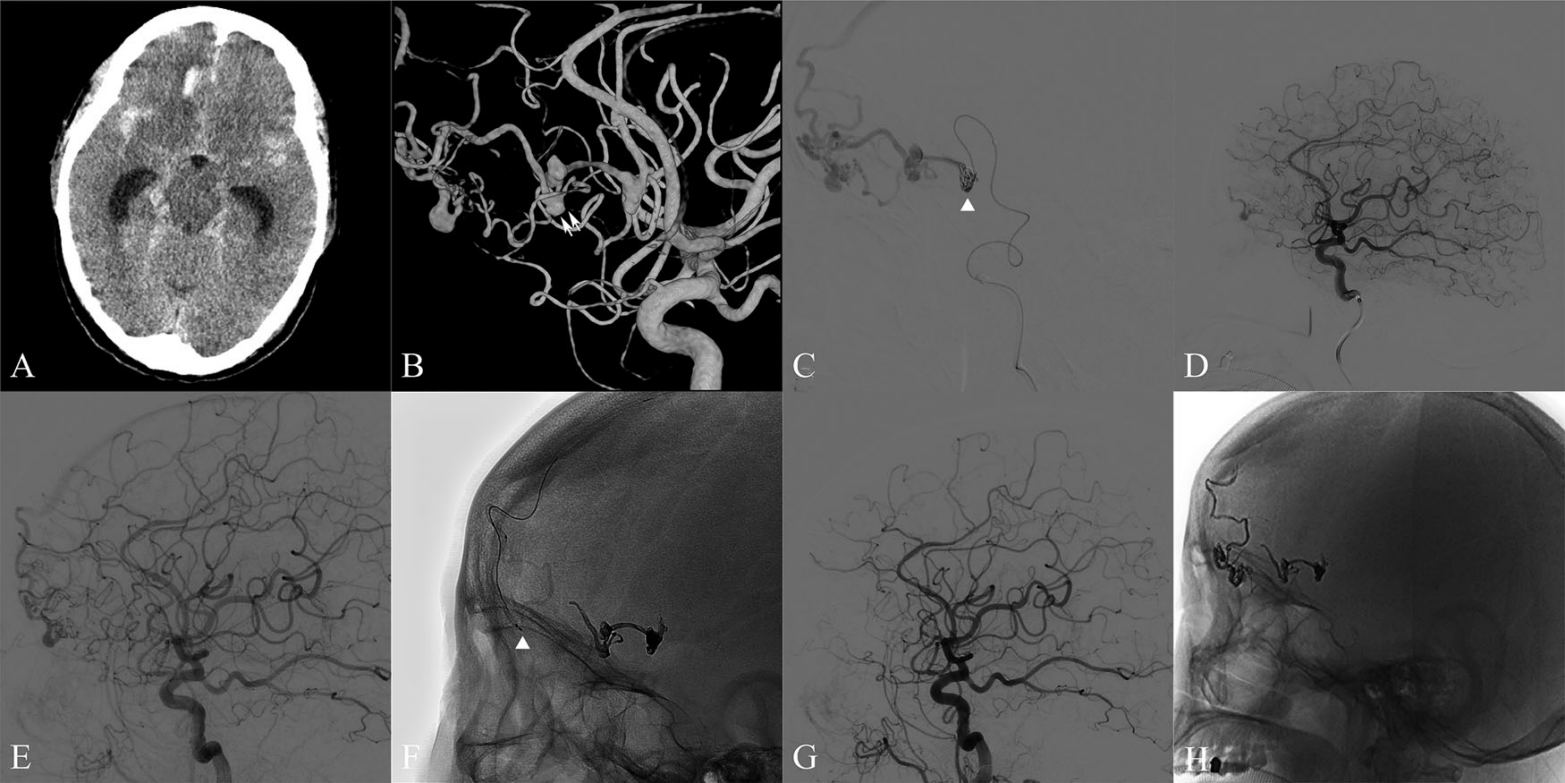
The patient was admitted to the emergency department with a sudden severe headache. Initial non‐contrast CT revealed extensive subarachnoid hemorrhage (A). Lateral right internal carotid angiogram with 3D reconstruction demonstrated a frontal base vascular malformation with flow‐related aneurysms on the orbitofrontal artery (B). Multiple tandem flow‐related aneurysms along the orbitofrontal artery were demonstrated by microcatheter angiography, with the largest one located at its origin (white arrow head) (C). Endovascular treatment of the aneurysms was performed using two coils and subsequent injection of 1.2 mL Onyx‐18. Post‐procedure angiogram confirmed complete aneurysm occlusion with decreased AVM flow (D). At planned second‐stage intervention 40 days later, a lateral right internal carotid angiogram revealed increased AVM flow with a partially obscured nidus (E). Non‐subtracted imaging demonstrated the Onyx cast from the initial procedure, and Dual Echelon‐10 microcatheters were navigated through the superior sagittal sinus to access the cortical veins (white arrow head) (F). Following deployment of two coils in the proximal draining vein, 1.8 mL Onyx‐18 was administered. Final lateral right internal carotid angiogram confirmed complete AVM obliteration (G). Non‐subtracted image showed the final Onyx cast after the second intervention (H).

**FIGURE 3:**
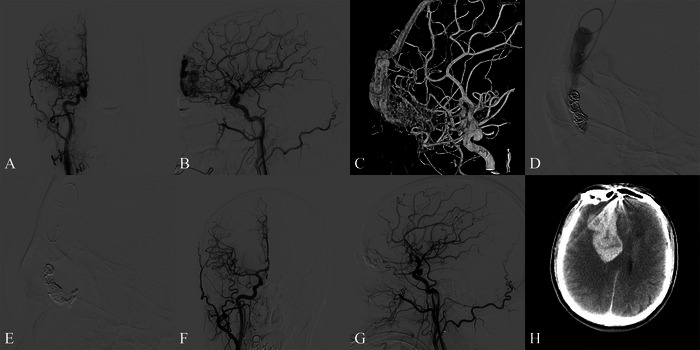
The patient was incidentally diagnosed with an intracranial vascular malformation during routine medical examination. Anteroposterior (A) and lateral (B) views of the right internal carotid artery angiogram revealed a frontal base MPD‐AVM supplied by both the anterior ethmoidal artery and the orbitofrontal artery. 3D reconstruction clearly demonstrated the nidus formation with tortuous and dilated draining veins (C). Transvenous embolization was performed, with microcatheter navigation through the superior sagittal sinus to access cortical veins, followed by deployment of two coils (D). Using the retrograde pressure cooker technique (RPCT), 1.2 mL of Onyx was injected (E). Immediate post‐procedure angiograms showed complete AVM obliteration (F, G). The patient subsequently experienced a severe hemorrhagic event, with CT revealing a massive right frontal lobe hematoma accompanied by contrast extravasation (H).

## Supporting information




**Supplementary Materials**: brb371099‐sup‐0001‐SuppMat.docx

**Supplementary Table S2**: brb371099‐sup‐0002‐TableS2.docx

## Data Availability

The data that support the findings of this study are available on request from the corresponding author. The data are not publicly available due to privacy or ethical restrictions.
