# Supplementary material for: Frontal Base Mixed Pial‐Dural Arteriovenous Malformations: A Distinct Entity Requiring Differentiated Treatment From Anterior Cranial Fossa Dural Arteriovenous Fistulas
Source: Brain Behav. 2025 Dec 7;15(12):e71099. doi: 10.1002/brb3.71099 (PMC12682932; doi:10.1002/brb3.71099)

|  |
| --- |

**Table S1 Baseline Characteristics and Clinical Features of Patients with ACF-DAVFs**

| **No.** | **Initial Symptoms** | **Hemorrhage** | **Cognard** | **Venous Aneurysm** | **Feeders** | **Venous Drainage** | **Approach** | **Route** | **Coil** | **Onxy** | **IAR** | **Complications** |
| --- | --- | --- | --- | --- | --- | --- | --- | --- | --- | --- | --- | --- |
| 1 | Headache, Dizziness | Yes | IV | Yes | Bi-AEA | CV | TAE | Lt-AEA | 0 | 0.8ml | Co | Thromboembolism |
| 2 | Headache | Yes | IV | Yes | Bi-FA | CV | TVE | SSS-CV | 2 | 2.5ml | Co. | None |
| 3 | Headache | No | IV | Yes | B1-AEA | CV | TVE | SSS-CV | 0 | 2.0ml | Co. | None |
| 4 | Diplopia, Dizziness | No | Ⅲ | No | Lt-AEA | CV | TVE | SSS-CV | 0 | 1.0ml | Co | None |
| 5 | Dizziness | No | Ⅲ | No | Bi-AEA | CV&DV | TAE | Lt-AEA | 0 | 1.2ml | InCo. | None |
| 6 | Syncope | No | IV | Yes | Rt-AEA & Rt-ECA | CV | TAE | Rt-AEA | 0 | 0.8ml | Co | None |
| 7 | Headache | No | Ⅲ | No | Bi-AEA | CV | TAE | Lt-AEA | 0 | 0.5ml | Co | None |
| 8 | Headache | Yes | IV | Yes | Lt-AEA | CV | TAE | Lt-AEA | 0 | 0.8ml | Co | None |
| 9 | Dizziness | Yes | Ⅲ | Yes | Bi-AEA | CV | TAE | Bi-AEA | 0 | 2.3ml | InCo. | None |
| 10 | Headache | Yes | Ⅲ | Yes | Rt-AEA | CV | TAE | Rt-AEA | 0 | 0.4ml | Co | None |
| 11 | Dizziness | No | Ⅲ | No | Bi-AEA & Lt-MMA | CV | TVE | SSS-CV | 0 | 0.8ml | Co. | None |
| 12 | None | No | IV | No | Bi-AEA | CV | TAE | Rt-AEA | 0 | 4.0ml | Co. | None |
| 13 | Headache | No | III | Yes | Bi-AEA & Bi-MMA | CV | TVE | SSS-CV | 2 | ml | Co. | None |
| 14 | Headache | Yes | III | No | Bi-AEA | CV | TVE | SSS-CV | 2 | ml | Co. | None |
| 15 | Headache | Yes | IV | Yes | Bi-ICA & Bi-ECA | CV | TVE | SSS-CV | 0 | ml | Co. | None |
| 16 | None | No | IV | Yes | Bi-AEA & Rt-IMA &  Bi-STA | CV | TAE | Bi-AEA | 1 | ml | InCo. | None |
| 17 | Dizziness | No | IV | No | Bi-AEA | CV & DV | TVE | SSS-CV | 1 | ml | Co. | None |
| 18 | None | No | III | No | Bi-AEA & Bi-IMA | CV | TVE | SSS-CV | 1 | ml | Co. | None |
| 19 | None | No | III | No | Rt-AEA & Lt-ICA &  Bi-ECA | CV | TAE | Rt-AEA | 0 | ml | Co. | Catheter Fracture |
| 20 | Dizziness | Yes | IV | Yes | Bi-ICA & Bi-ECA | CV | TVE | SSS-CV | 5 | ml | Co. | None |
| 21 | Headache,  Dizziness | Yes | IV | Yes | Bi-AEA | CV | TVE | SSS-CV | 0 | ml | Co. | None |
| 22 | Headache,  Dizziness | Yes | IV | Yes | Bi-AEA | CV | TVE | SSS-CV | 0 | ml | Co. | None |
| 23 | Dizziness | Yes | IV | Yes | Bi-AEA & Bi-STA | CV | TVE | SSS-CV | 2 | ml | Co. | None |
| 24 | Dizziness | No | IV | Yes | Bi-AEA & Lt-MMA | CV | TVE | SSS-CV | 0 | ml | Co. | None |
| 25 | Headache,  Dizziness | Yes | IV | Yes | Bi-AEA | CV | TAE | Bi-AEA | 0 | ml | Co. | None |
| 26 | Proptosis,  Chemosis | No | III | No | Bi-AEA | Lt-SOV | TVE | IPS | 0 | ml | Co. | None |
| 27 | None | No | IV | Yes | Bi-AEA | CV | TAE | Lt-AEA | 0 | ml | Co. | None |
| 28 | Headache | No | IV | No | Bi-AEA | CV | TAE | Bi-AEA | 0 | ml | InCo. | None |
| 29 | Headache | Yes | IV | Yes | Bi-AEA & Bi-IMA | CV | TAE | Lt-AEA | 0 | ml | Co. | None |

Rt: Right; Lt: Left; Bi: Bilateral; ICA: Internal Carotid Artery; ECA: External Carotid Artery; AEA: Anterior Ethmoidal Artery; OFA: Orbitofrontal Artery; IMA: Internal Maxillary Artery; STA: Superficial Temporal Artery; MMA: Middle Meningeal Artery; SSS: Superior Sagittal Sinus; SOV: Superior Ophthalmic Vein; IPS: Inferior Petrosal Sinus; CV: Cortical Vein; DV: Deep Vein; IAR: Immediate Angiographic Results; InCo.: Incomplete; Co.:Complete; GCS: Glasgow Coma Scale; mRS: modified Rankin Scale; Pre.:Preoperative; Post: Postoperative.

**Figure S1 Patient Grouping and Clinical Management Status**


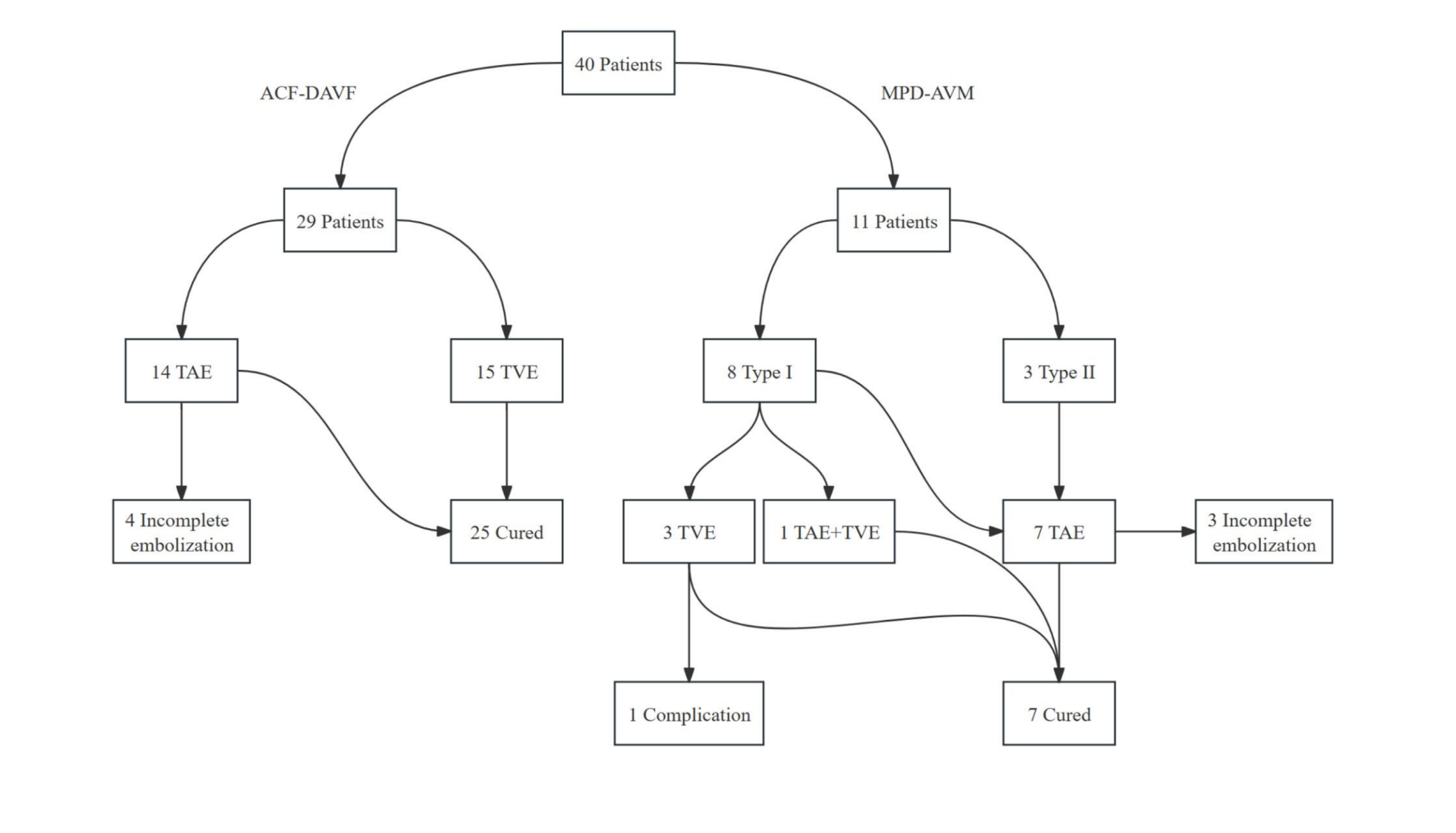

Supplement: Supplementary file 1 — Supplementary Materials: brb371099‐sup‐0001‐SuppMat.docx [file BRB3-15-e71099-s002.docx]
